# The Dangerous Drugs Act Regulations

**Published:** 1921-01-22

**Authors:** 


### THE DANGEROUS DRUGS ACT REGULATIONS.

We have previously outlined the provisions of the Dangerous Drugs Act, 1920, and drew attention to the fact that pharmacists in retail business were made "authorised persons" to have in their possession and supply, under certain conditions, substances mentioned in the Act, whereas no express provision was made covering pharmacists engaged in dispensing at public hospitals and institutions. The Regulations have now been published in draft form, and we predict that, if passed without amendment, the practice and customs of supplying medicinal preparations containing the drugs will be revolutionised. The drugs to which the Act applies are cocaine, ecgonine, morphine, diamorphine (or heroin), medicinal opium, and any preparation or admixture of these drugs containing not less than $\frac{1}{5}$ per cent. of morphine or $\frac{1}{10}$ per cent. of cocaine, ecgonine, or diamorphine.

The very wide field which this covers is to be noted. For instance, practically *all* morphia preparations, such as tincture of opium, Dover's powder, gall and opium ointment, injection tablets, etc., may not be supplied except on a prescription signed by a registered medical practitioner with his full name, qualifications, address, and the words "Not to be repeated"; the prescription, too, must be retained in the premises (dispensary) where it is dispensed, and, finally, an entry of every supply must be recorded in books kept for the purpose solely. A separate book must be kept for each drug, so that five books will be required!

All of the drugs bought must be entered up in its appropriate book, both by the wholesale drug supplier and the hospital pharmacist who receives the goods.

No indication is given in these elaborate Regulations as to the procedure to be adopted in recording the buying or sale of preparations containing two or more of the drugs, and we suggest that, for hospital practice, *one* book—such as the usual stock book or ledger denoting drugs bought—ought to suffice. We propose to give briefly the particular clauses which are likely to affect hospital work.

No person may be in possession of the drugs, unless they can prove these were supplied on the order of a medical practitioner; but the Secretary of State may grant an exemption from this Regulation in respect of the dispensing of the drugs at a public hospital.

It would appear that this clause is intended to allow, say, a ward sister to have in her possession supplies of the drugs for use as ordered by the medical officer; it would obviously be ridiculous to have to send to the dispensary for every dose of morphia injection required.

The details required from medical practitioners ordering prescriptions at hospitals are absolutely unworkable, and we suggest that bed cards should be accepted under the Act as "prescriptions," initialled by the medical officer in charge; these are always filed, and whilst they would not be retained in the actual dispensing premises, they could easily be referred to if required. The records to be kept by the hospital pharmacist, if insisted on, will prove very onerous and necessitate clerical assistance in the dispensary.

Again, whilst a retail pharmacist may manufacture any preparation or admixture of the drugs, the hospital pharmacist, apparently, has no power to do this, but only to be in possession of and supply the drugs.

The position created is quite preposterous, and would practically mean that all preparations covered by the Act would have to be bought ready-made. What would become of the pharmaceutical laboratories at our large hospitals which contribute so largely to supply preparations of guaranteed efficiency at reduced cost?

Paragraph 13 of the Regulations would render it necessary for a hospital requiring supplies of the drugs to send a messenger with a signed order authorising him to receive the goods.

A hospital, say at Cambridge, requiring urgently a morphia preparation could not wire or telephone to the London wholesaler for it to be sent by post or train, even if a confirmatory written order was to follow by post.

The Regulations have been designed to stop illegal doping, but we suggest that there is no evidence that supplies have been obtained illicitly from hospitals, and the imposition of such records which the Regulations demand will add to the financial burden of our hospitals and hinder legitimate and urgent use of the drugs.

It would be a good plan if the Home Office, even at this stage, would set up an Advisory Committee of Hospital and Institution Pharmacists to make recommendations as to the application of the Regulations to public hospitals and institutions.

Unless altered, the Regulations come into force forty days after January 7, 1921.

